# Highly sensitive biomolecular interaction detection method using optical bound/free separation with grating-coupled surface plasmon field-enhanced fluorescence spectroscopy (GC-SPFS)

**DOI:** 10.1371/journal.pone.0220578

**Published:** 2019-08-01

**Authors:** Takatoshi Kaya, Satoru Nagatoishi, Kosuke Nagae, Yukito Nakamura, Kohei Tsumoto

**Affiliations:** 1 Corporate R&D Headquarters, Konica Minolta, Inc., Hino-shi, Tokyo, Japan; 2 Institute of Medical Science, the University of Tokyo, Tokyo, Japan; 3 Department of Bioengineering, School of Engineering, the University of Tokyo, Hongo Bunkyo-ku, Tokyo, Japan; VIT University, INDIA

## Abstract

Grating-coupled surface plasmon field-enhanced fluorescence spectroscopy (GC-SPFS) with optical bound/free (B/F) separation technique was developed by employing a highly directional fluorescence with polarization of surface plasmon-coupled emission (SPCE) to realize highly sensitive immunoassay regardless of the ligand affinity. A highly sensitive immunoassay system with GC-SPFS was constructed using a plastic sensor chip reproducibly fabricated in-house by nanoimprinting and applied to the quantitative detection of an anti-lysozyme single-domain antibody (sdAb), to compare conventional washing B/F separation with optical B/F separation. Differences in the affinity of the anti-lysozyme sdAb, induced by artificial mutation of only one amino acid residue in the variable domain were attributed to higher sensitivity than that of the conventional Biacore surface plasmon resonance (SPR) system. The detection limit (LOD; means of six replicates of the zero standard plus three standard deviations) of the GC-SPFS immunoassay with optical B/F separation, was estimated to be 1.2 ng/ml with the low-affinity ligand (mutant sdAb Y52A: K_D_ level was of the order of 10^−7^ ~ 10^−6^ M) and was clearly improved as compared to that (LOD: 9.4 ng/ml) obtained with the conventional washing B/F separation. These results indicate that GC-SPFS with the optical B/F separation technique offers opportunities to re-evaluate low-affinity biomaterials that are neither fully utilized nor widespread, and could facilitate the creation of novel and innovative methods in drug and diagnostic development.

## Introduction

In recent years, various biomaterials for drug development, including cancer immunity drugs [1] and anti-drug antibodies (ADAs) [2–4], have rapidly gained importance in next-generation precision medicine [5]. These biomaterial drugs are less stable than conventional antibody drugs due to their different IgG subclasses and modifications. Additionally, an understanding of aggregation by biomaterial drug components including non-proteins is essential as almost all biomaterial drugs consist of proteins with concentrations reaching 100 mg/ml [6–8]. Thus, suitable quality control is required for the safe use of biopharmaceuticals [9]. Evaluation requires high sensitivity for a wide range of binding affinities, with reconfirmation of conventional technology and various types of analytical technique in different areas [9].

In response to the above, various real-time bioanalytical techniques including established methods, are used to analyze biomaterials including proteins [10–14]. An SPR system is critical for evaluating various biomaterial drugs; consequently, this has become indispensable for discovering various drugs. Since SPR can measure real-time interactions at concentrations below one nanogram per milliliter with no labeling [14–16], the Biacore SPR system has become a widespread standard method for validating and/or evaluating various biomaterials in drug companies. Additionally, a quartz crystal microbalance (QCM) gives highly time-resolved information on specific and non-specific protein interactions [17]. For evaluation with a QCM, various coatings on gold layers are available, while there is a limited choice for SPR evaluation. As the QCM signal is strongly affected by the viscoelasticity of the biological substance, it is unsuitable for measuring soft biological materials. Although labeling is not required for SPR and QCM, it is almost impossible to distinguish between specific and non-specific interactions with no control or reference test. Additionally, the sensitivity limitations of SPR and QCM technologies require preparation of approximately 100 μl sample with a concentration of about 1~10 ng/ml when using a high-affinity ligand material. Additionally, affinity analysis of extremely small or large molecules with weak or soft interactions by SPR and QCM methods is unpopular due to their insufficient sensitivity and the intrinsic limitations of their detection methods.

**Fig 1:**
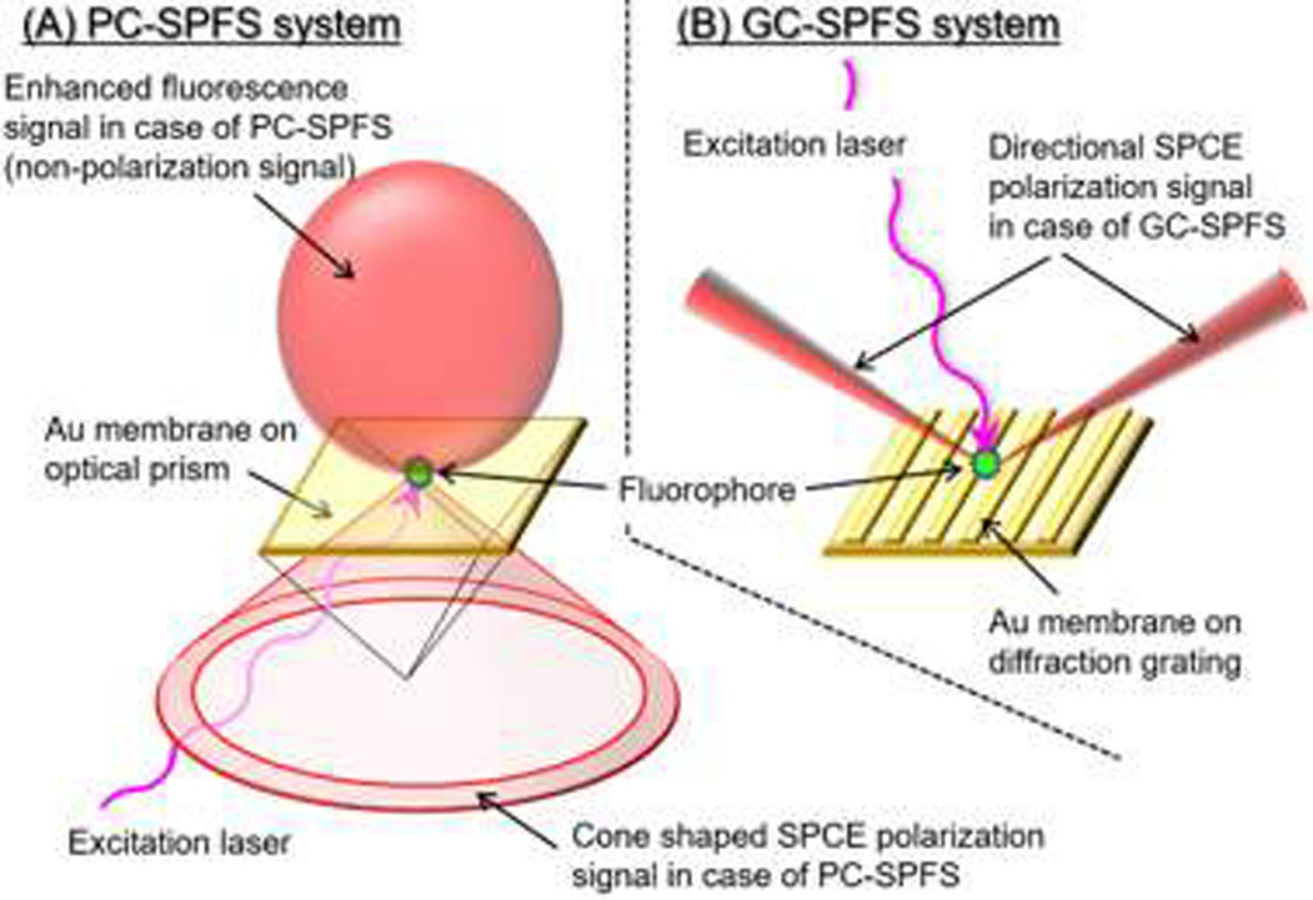
Schematic views of principle of surface plasmon coupled emission (SPCE). (A) SPCE in prism-coupled surface plasmon field-enhanced fluorescence spectroscopy (PC-SPFS) and (B) SPCE in grating-coupled surface plasmon field-enhanced fluorescence spectroscopy (GC-SPFS). SPCE integrates fluorescence and plasmonics, and the SPCE signal has direction and polarization. The directional SPCE polarization signal in GC-SPFS is much higher than cone-shaped SPCE polarization signal in PC-SPFS.

Surface plasmon field-enhanced fluorescence spectroscopy (SPFS) is a highly sensitive fluorescence detection method compared with conventional fluorescence techniques and uses the SPR phenomenon. SPFS was developed by several groups and is expected to have a sensitivity of a single pg/ml or less for analyzing biomaterial interactions and immunoassays [18–20]. Bernhagen et al. measured affinity binding constants (K_D_) values between Cy-5 labeled peptide and integrin receptor by using SPFS [21]. Furthermore, a highly sensitive SPFS immunoassay system employing a disposable plastic prism sensor has been developed, and the cutting-edge clinical performance of a prostate-specific antigen glycosylation isomer for discriminating between prostate cancer and benign prostatic hyperplasia has been reported [22,23]. SPR technology is broadly classified into prism-coupled SPR (PC-SPR; Fig 1A) and grating-coupled SPR (GC-SPR; Fig 1B) systems [24,25]. Biacore and other companies have adopted PC-SPR to achieve a stable system performance. However, GC-SPR is uncommon because of the need to fabricate reproducibly a diffraction grating. However, GC-SPR has several advantages; in particular, it has a simple optical setup that, unlike PC-SPR, does not require a prism. Some studies of GC-SPR excited fluorescence were already reported in the field of application to the organic light-emitting diodes [26,27]. Tawa et al. fabricated a metal-coated diffraction grating with simple nanoimprinting technology, which they termed a “plasmonic chip,” and used this structure for immunosensing applications [28]. Furthermore, this structure could be used to achieve highly sensitive detection by using surface plasmon-coupled emission (SPCE; Fig 1A and 1B) [29]. The SPCE technique, initially described by Lakowicz et al., successfully combines fluorescence and SPR by employing the prism-coupled configuration (Fig 1A) [30–34]. The sensitivity can be significantly increased, and the unique properties of this approach are useful for developing novel analytical methods. Although the detection sensitivity is insufficient for a bioaffinity analysis and there are no reproducible measurements in the literature, optical B/F separation was demonstrated for the first time by using the directional properties of SPCE in the prism-coupled condition system [31].

In this study (S1 Fig), we initially theoretically investigate the SPCE polarizability of grating-coupled surface plasmon field-enhanced fluorescence spectroscopy (GC-SPFS) fluorescence signals from an Au-coated diffraction grating surface (Fig 1B), which was reproducibly fabricated in-house. Additionally, we demonstrate real-time bioaffinity analysis with GC-SPFS using an SPCE signal with high polarizability and narrow directionality by employing the anti-lysozyme single-domain antibody (sdAb) as a high-affinity biomaterial model together with an anti-lysozyme sdAb mutant as a low-affinity biomaterial model. Moreover, we also evaluate a GC-SPFS immunoassay with no washing process for B/F separation by using the SPCE phenomenon and verify that low-affinity biomaterials, which are difficult to measure using conventional immunoassay methods, can be measured with high sensitivity.

## Materials and methods

### Materials

Allophycocyanin (Dojindo Molecular Technologies, Inc., Kumamoto, Japan) and lysozyme (Wako Pure Chemical Industries Ltd., Japan) were used as received. *N*-hydroxysuccinimide (NHS) and 1-ethyl-3-(3-dimethylaminopropyl) carbodiimide hydrochloride (EDC) were obtained from Wako Pure Chemical Industries, Ltd. (Osaka, Japan).

### Wild-type anti-lysozyme sdAb and Y52A mutant antibody

The gene encoded sdAb amplified by a polymerase chain reaction was inserted into a pRA2 vector. Using the *Escherichia coli* BL21 (DE3) expression system (Merck, Darmstadt, Germany), we derived a recombinant sdAb as a soluble protein. BL21 (DE3) cells carrying the appropriate expression plasmid were precultured in 3 ml of the LB medium with 50 mg/l ampicillin overnight at 37°C. The precultured cells were then inoculated into 1000 ml of the LB medium containing 50 mg/l ampicillin and shaken at 37°C until the optical density at 600 nm reached 0.6. Isopropyl β-D-thiogalactopyranoside was added to a final concentration of 0.5 mM, and the mixture was shaken at 20°C for 15–20 h. Cells were harvested by centrifugation at 7000 g for 10 min at 4°C, and the pellet thus obtained was resuspended in 50 ml of a solution containing 50 mM Tris-HCl (pH 8.0) and 500 mM NaCl (buffer A). The cells were sonicated using an ultrasonic cell-disruptor (Tommy, Tomigusuku, Japan) for 15 min (Output 7, Duty 50) and then centrifuged at 40,000 g for 30 min. The soluble solution was applied to a Ni-NTA column (Novagen, Takara, Japan) equilibrated with buffer A containing 5 mM imidazole. The protein was eluted with a stepwise increase in the imidazole concentration (10, 20, 50, 100, 200, and 500 mM) in buffer A. The eluent was purified by size-exclusion chromatography (Hiload 26/60 Superdex 75 pg, GE Healthcare, Uppsala, Sweden). The purity of the sdAb and its mutein was verified by sodium dodecyl sulfate-polyacrylamide gel electrophoresis and by the ratio of the UV absorbance at 260 nm to that at 280 nm (less than 0.65). The concentrations of the wild type and Y52A sdAb were determined by the molecular absorption coefficient 24410 and 22920 at 280 nm, respectively.

### Preparation of the GC-SPFS sensor chip

**Fig 2:**
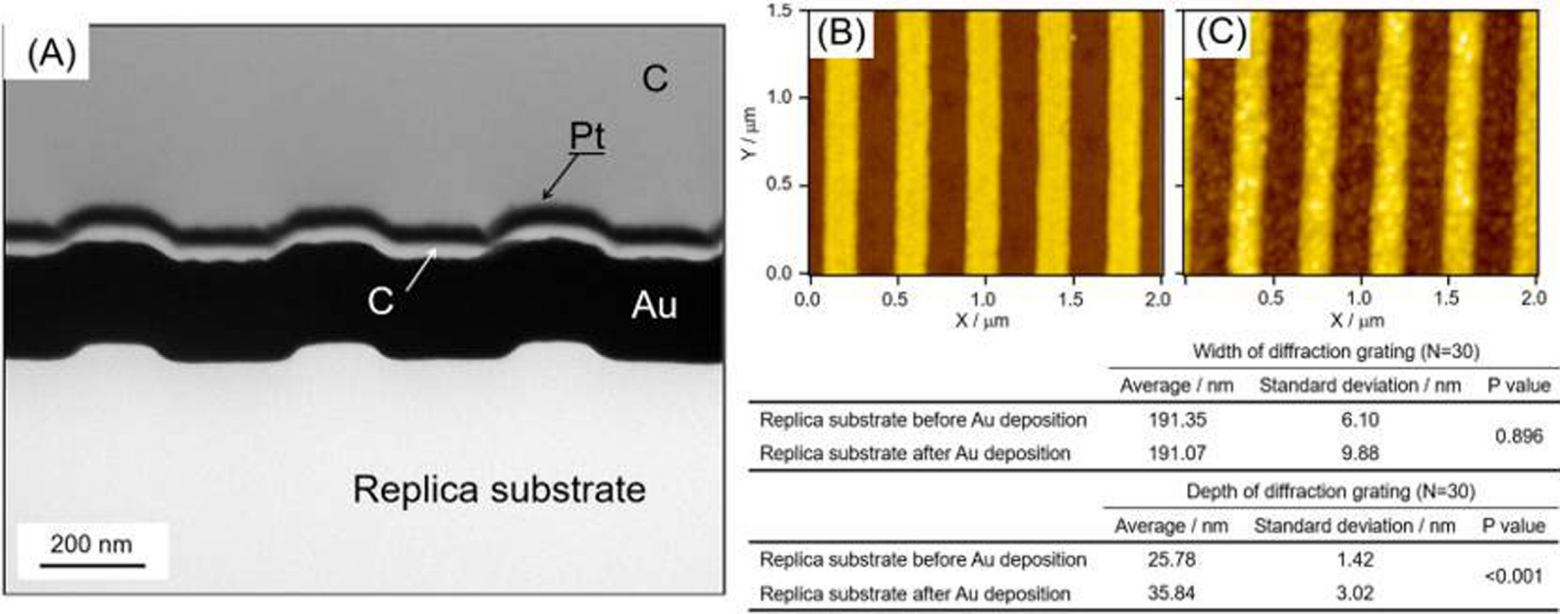
AFM images of diffraction grating nanoimprinting replica substrate. (A) SEM image of a cross-section of the surface of the GC-SPFS sensor chip. This was acquired from a pre-coated GC-SPFS sensor chip of carbon/Pt/carbon for clear observation. The pitch, depth, and duty ratio (ratio of the convex surface of the pitch) of the grating design were 400 nm, 20 nm, and 0.50, respectively.AFM images of diffraction grating nanoimprinting replica substrate before (B) and after (C) Au deposition. Reproducibility evaluation by AFM as shown in the table, indicates that there was no change in the width of the diffraction grating after Au deposition. However, the depth of the diffraction grating and its surface Ra were clearly changed after Au deposition.

We first prepared a silicon master mold by electron beam drawing (F7000S, Advantest, Inc., Tokyo, Japan), and a replica of this was created using poly(dimethylsiloxane) (PDMS) (SILPOT 184W/C, Toray Inc., Tokyo, Japan) as a secondary mold. The grating master design had a pitch and depth of 400 and 20 nm, respectively. We fabricated a diffraction grating replica substrate by using the secondary mold as a GC-SPFS disposable plastic sensor chip on the plastic substrate (PMMA, 2 mm thick). The diffraction grating was prepared in-house using a UV nanoimprinting technique with a photocurable resin (LU1106HA, Daicel Corporation, Osaka, Japan), as mentioned earlier [28,29]. A gold membrane with a thickness of approximately 180 nm was prepared on the diffraction grating replica substrate by magnetron sputtering (L-430S-FHS, Canon Anelva Corp., Kanagawa, Japan). An SEM image of a cross-section of the grating diffraction substrate after gold deposition is shown in Fig 2A. The fabrication reproducibility of the diffraction grating width and depth were evaluated for six replica substrates (five points in one substrate) by atomic force microscopy (AFM; SPA400/NanoNaviII, Hitachi High-Tech Science Corporation, Tokyo, Japan) before (Fig 2B) and after (Fig 2C) gold deposition. The grating structure evaluation (*N* = 30) from AFM images before and after gold deposition clearly showed that the coefficient of variation was below 10% before and after gold deposition with respect to the width and depth of the diffraction grating structure. There was no change in the average of width of the diffraction grating after Au deposition (*p* = 0.896). However, the average depth of the diffraction grating and its surface roughness (Ra) were clearly changed after gold deposition (*p* < 0.001). We then fabricated a self-assembled monolayer (SAM) using an amine-terminated thiol (11-amino-1-undecanethiol, hydrochloride; Dojindo Molecular Technologies Inc., Kumamoto, Japan) on the gold membrane of a diffraction grating replica substrate. Carboxymethyl dextran (CMD; Meito Sangyo Co. Ltd., Nagoya, Japan) and lysozyme or allophycocyanin were then sequentially immobilized via an amide coupling reaction (100 mM NHS and 100 mM EDC) on the gold membrane using SAM and CMD [22]. Finally, a plastic substrate (PMMA, 2 mm thick) with an inlet and outlet was fixed with black double-sided adhesive tape to a plastic substrate after completing the blocking step using 1% bovine serum albumin (BSA)-PBS (Blocker BSA in 10× PBS; Pierce) after immobilizing the lysozyme or allophycocyanin. The bonding process produced a microchannel (50 μm high, 2.4 mm wide, and 20 mm long) between the cover glass plate and the plastic substrate.

### Optical setup of GC-SPFS

**Fig 3:**
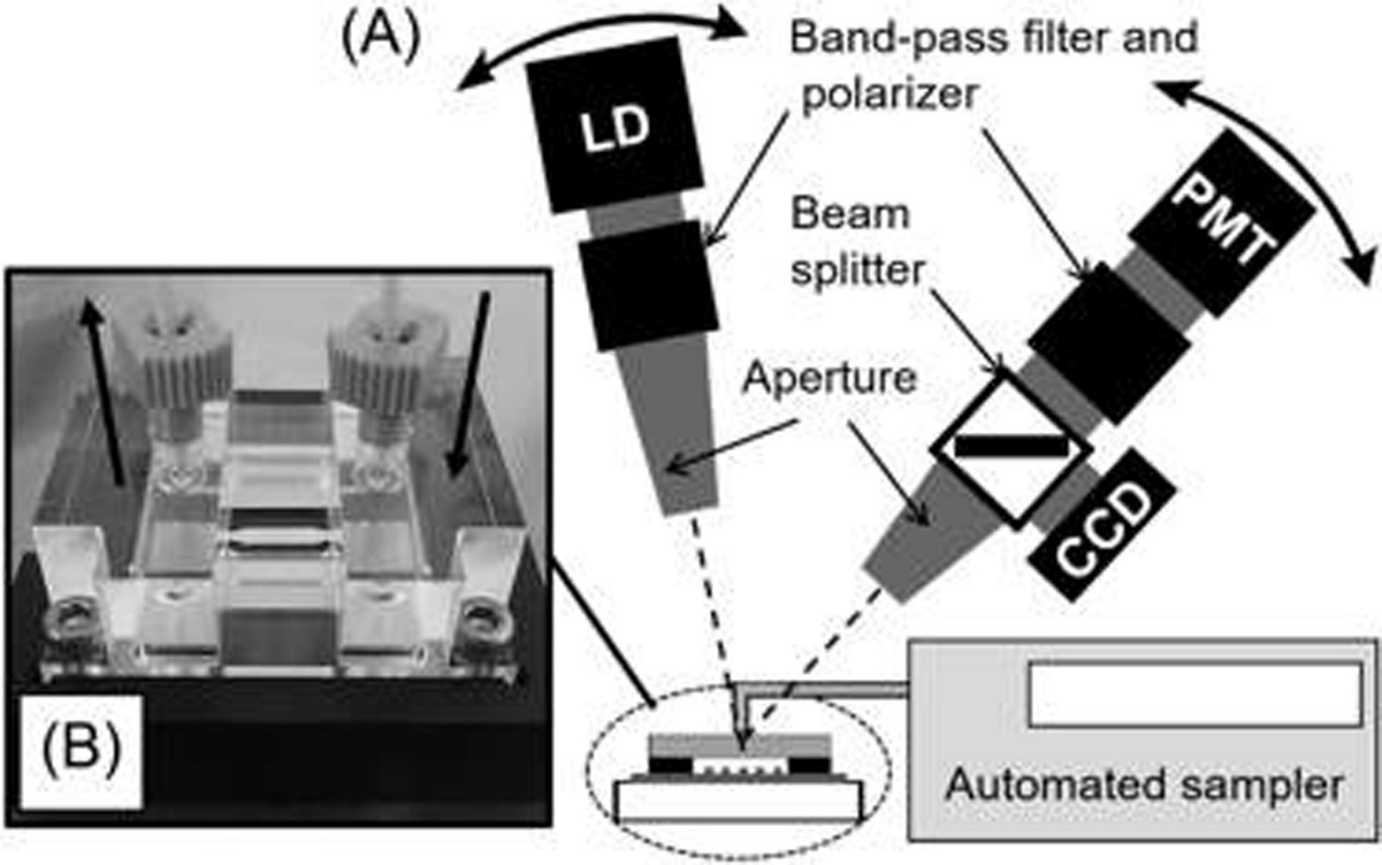
Optical setup of the GC-SPFS system. (A) Optical setup of the GC-SPFS system developed in-house. This employs a microchannel flow system for affinity analysis and quantitative immunoassay. (B) Image of the GC-SPFS sensor chip connected to the microchannel flow system.

The optical setup of GC-SPFS system is shown in Fig 3A [29]. A collimated laser beam from a laser diode (HL6322G, Opnext Inc., Eatontown, CA, USA), with a wavelength of 637 nm, was passed through a band-pass filter (DIF-BPF-2 (half-width; 630 ± 8 nm), Optical Coatings Japan, Tokyo, Japan) and polarizer (USP-20C-01, Sigma Koki, Saitama, Japan). The incident light was p-polarized with a power of 10 μW, and the illuminated area was a spot 1 mm diameter on the grating substrate surface. The diode laser was mounted on a rotational arm, allowing the incident light to be varied. A GC-SPFS sensor chip was placed horizontally on the sample stage at the rotational center. The light reflected from the grating surface substrate was monitored using a CCD camera (STC-MB33USB, Sentech Co. Ltd., Kanagawa, Japan) through a long cylindrical aperture mounted on a second arm. The fluorescence signal was measured with a photomultiplier tube (PMT) (H7421-40, Hamamatsu Photonics K.K., Shizuoka, Japan) mounted on the same arm as the CCD, and a polarizer (USP-20C-01, Sigma Koki, Saitama, Japan) and an emission filter (DIF-BPF-1 (half-width; 668 ± 5 nm), Optical Coatings, Tokyo, Japan) were inserted in front of the PMT. The incident angle was fixed at the resonance angle, and the fluorescence intensity was measured as a function of the detection angle with the PMT mounted on the second rotational stage.

### Affinity analysis and immunoassay with GC-SPFS

A polarizing analysis with allophycocyanin and a GC-SPFS affinity analysis were automatically carried out by the GC-SPFS measurement setup and microchannel flow system (Fig 3B). A GC-SPFS sensor chip, which was prepared by immobilizing allophycocyanin or lysozyme, was mounted on the chip stage and connected directly to the microchannel flow system through a PEEK tube and tube connector, both of which were obtained from Shimadzu GLC Ltd., Tokyo, Japan. Allophycocyanin and the sdAb (wild-type sdAb and Y52A mutant) were diluted in 1%BSA-PBS as binding buffer or sample dilution buffer. All the solutions, which including the loading buffer and washing buffer (Tris-buffered saline with Tween 20, TBS-T, Wako Pure Chemical Industries Ltd., Osaka, Japan), were continuously injected at the rate of 20.0 μl/min. Incorporation of two air bubbles between the buffer and measurement sample prevented solution mixing to achieve an accurate microflow analysis. A polarization analysis with GC-SPFS was conducted both with and without 0.2 nM Alexa-647 fluorescent dye in the measurement solution. During the lysozyme affinity assay, 100 μl of the measurement sample containing wild-type anti-lysozyme sdAb and Y52A mutant was left to react for 5.0 min in the GC-SPFS sensor chip.

In this study, GC-SPFS immunoassay signals were measured using p- and s-polarized fluorescence and were collected by 90° rotation of the polarizer in front of the PMT before injecting washing buffer. During washing buffer injection, GC-SPFS immunoassay signals were then collected only p-polarized fluorescence for evaluation of conventional washing B/F separation. The GC-SPFS immunoassay of the lysozyme was evaluated using two measurement modes: the optical B/F separation mode corrected at 300 sec, and the conventional washing B/F separation mode corrected at 730 sec from GC-SPFS real-time affinity signal curves for different concentrations (36 pg/ml–150 ng/mL) of the Alexa-647-labeled wild-type anti-lysozyme sdAb and Y52A mutant. The incident resonance angle and receiving angle were obtained as optimum conditions for GC-SPFS accurate analysis for each measurement. All the affinity assays were performed at the same incident resonance angle (8.0°–9.0°) and optimum receiving angle (14.5°–15.0°) at room temperature. A GC-SPFS affinity analysis was performed to calculate the dissociation constants using the 1:1 Langmuir binding mode and BIA evaluation software (version 3.0, GE Healthcare Bio-Sciences AB, Uppsala, Sweden).

### Affinity analysis by using a Biacore system

The binding affinities for the wild-type sdAb and Y52A mutant of the lysozyme were measured by SPR assays using a Biacore 2000 system. Lysozyme antigen was immobilized on a CM5 sensor chip (GE Healthcare Bio-Sciences AB, Uppsala, Sweden) by the amide-coupling reaction using 100 mM NHS and 100 mM EDC. The coupling densities were controlled at 2000 RU. The wild-type sdAb and Y52A mutant were diluted in an HBS-EP (0.01 M HEPES-NaOH, pH 7.4, 150 mM NaCl, 0.005% v/v polysorbate-20 [GE Healthcare Bio-Sciences AB]) buffer and injected into the sensor chip for 5 min at a rate of 20 μl/min. The reaction time was set as a condition sufficient for sdAb affinity analysis in this study. Different concentrations (4.6–150 ng/mL) of wild type sdAb and Y52A mutant were introduced to the lysozyme antigen-capture surfaces. All the affinity analyses were performed at 25°C, and the surface signals with the pure buffer were subtracted by calculating of the dissociation constants with the 1:1 Langmuir binding mode and BIA evaluation software (version 3.0, GE Healthcare Bio-Sciences AB).

### Lysozyme ELISA

ELISA was employed as a comparative assay method; lysozyme was coated onto 96-well plates (MaxiSorp F96, Thermo Fisher Scientific Inc., Franklin, MA, USA) at 0.25 μg/well at pH 9.6 in carbonate buffer and incubated overnight at 4°C. The wells were washed with PBS-T and before blocking for 2 h at room temperature with 100 μl of 1% BSA in PBS. Next, biotinylated wild-type sdAb and Y52A mutant were then prepared with a biotin-labeling kit (Dojindo Molecular Technologies Inc., Kumamoto, Japan); 50 μl of the biotinylated wild-type sdAb and Y52A mutant were diluted to final concentrations of 36 pg/ml to 2400 ng/ml with 1% BSA–PBS added to each well, and the plates were incubated for 1 h at room temperature. After washing the plates three times with PBS–T, 0.0125 μg/mL streptavidin-conjugated horseradish peroxidase (Thermo Fisher Scientific Inc.) in 1% BSA–PBS was added, and the plates were incubated for an additional 30 min. After further washing with PBS-T several times, a chemiluminescence substrate (SuperSignal ELISA Femto Substrate, Thermo Fisher Scientific Inc.) was added for 5 min at room temperature, and luminescence signals were measured with a microplate reader (SH-9000; Corona Electric Co. Ltd., Ibaraki, Japan).

## Results and discussion

### Polarization analysis of GC-SPFS fluorescence

**Fig 4:**
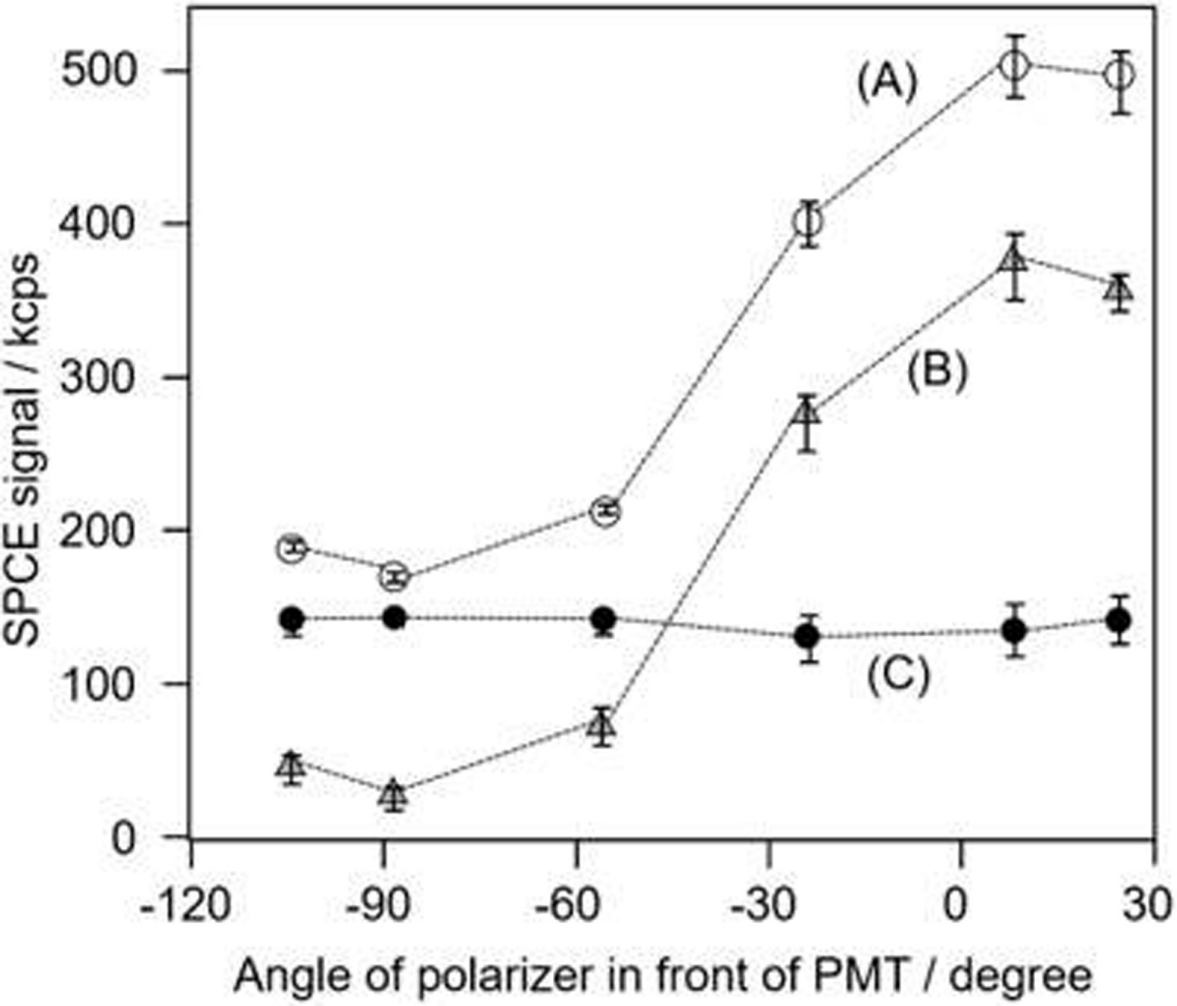
SPCE signal curves as a function of the polarization. Allophycocyanin SPCE signal curves as a function of the polarization angle of a polarizer placed in front of the PMT with (A) and without (B) Alexa-647 fluorescent dye in the measurement buffer solution. Curve (C) is the difference between curves (A) and (B). All of the points were measured three to five times.

The polarization specificity of the GC-SPFS allophycocyanin fluorescence signals based on SPCE was determined by using a polarizer in front of the PMT detector with and without fluorophore in the bulk solution, as shown in Fig 4. All points were measured at three to five times using one same sensor chip for reproducibility, and the origin of the horizontal axis indicates that the polarizer is orthogonal to the diffraction grating on the GC-SPFS sensor surface, i.e., the p-polarized position. Thus, the data shown in Fig 4B suggest that the GC-SPFS fluorescence signal was highly p-polarized under the conditions when the fluorophore was absent. We previously explored the angular distribution of the GC-SPFS fluorescence signal using the same optical setup and confirmed the narrow directionality of the fluorescence signal with Au and Ag as plasmonic materials [29]. Thus, the GC-SPFS fluorescence specificity with respect to the polarization and angular distribution [35,36] clearly confirm that the GC-SPFS fluorescence is SPCE. The SPCE phenomenon is described by Lakowicz et al. as being similar to SPR in reverse (Fig 1A) [30–34]. Moreover, the polarization of the SPCE signals from the metal grating was also measured with 0.2 nM Alexa-647 in the bulk solution, as shown in Fig 4A. The peak value of SPCE appeared at the same position as that with no fluorophore in the bulk solution. Furthermore, there is no clear difference between the polarization with and without Alexa-647 in the bulk solution, as shown in Fig 4C.

These results suggest that the fluorescence emitted from Alexa-647 in the bulk solution is directly excited by incident light with no effects from the surface-plasmon-enhanced field. Since SPCE is only induced when a fluorophore is near the metal-coated grating surface, the SPCE signal could be employed to measure the surface interaction with no bulk influence. Matveeva et al. investigated SPCE phenomena under the prism-coupling condition and measured both directional and polarized signals as for biosurface analysis applications [30]. The SPCE signal with prism coupling would radiate in the direction of a conical surface pass through the metal membrane prepared on the prism (Fig 1A) [34,37]. It is estimated that the SPCE signal for prism coupling is significantly decreased by optical shielding from the metal membrane, which is essential for inducing SPCE. Moreover, it is difficult to mechanically detect all angle of the widely spread SPCE signals from prism coupling without enlarging the system. Overall, SPCE cannot be efficiently utilized with prism coupling. However, SPCE from a metal grating is emitted only in two directions on the same side as both the emission light and solution; therefore, detection of the SPCE signal from a metal grating is considerably more efficient than from a prism, resulting in highly sensitive technology (Fig 1B) [35,36].

The SPCE signal from a bound fluorophore (*I*_*1*_) contains p- and s-polarized components (*I*_*p1*_ and *I*_*s1*_, respectively), while the noise from a bulk fluorophore (*I*_*2*_) also has both components (*I*_*p2*_ and *I*_*s2*_, respectively). The p- and s-components of the fluorescence emitted from a GC-SPFS chip can be measured by rotating the polarizer between the GC-SPFS sensor chip and the PMT detector according to the following equations.

$$I_{p}=I_{p1}+I_{p2},\;I_{s}=I_{s1}+I_{s2}$$ (1)

Fig 4C shows an unpolarized signal (*I*_*p2*_ is equal to *I*_*s2*_); thus, the difference between *I*_*p*_ and *I*_*s*_ is described as follows:

$$I_{p}-I_{s}=I_{p1}-I_{s1}+\left(I_{p2}-I_{s2}\right)=I_{p1}-I_{s1}$$ (2)

We estimated the difference between *I*_*p*_ and *I*_*s*_ to be the reaction response of the GC-SPFS sensor surface and applied this idea to affinity analysis and immunoassay with no conventional washing process. Matveeva et al. applied the angular distribution of the SPCE signal to affinity analysis without B/F separation in prism coupling mode [31]. In this work, we applied the SPCE specification of the angular distribution and polarization by employing a metal-coated grating surface to distinguish a sensor surface reaction from a bulk fluorophore as a new optical B/F separation method.

### Affinity evaluation with the Biacore and GC-SPFS systems

**Fig 5:**
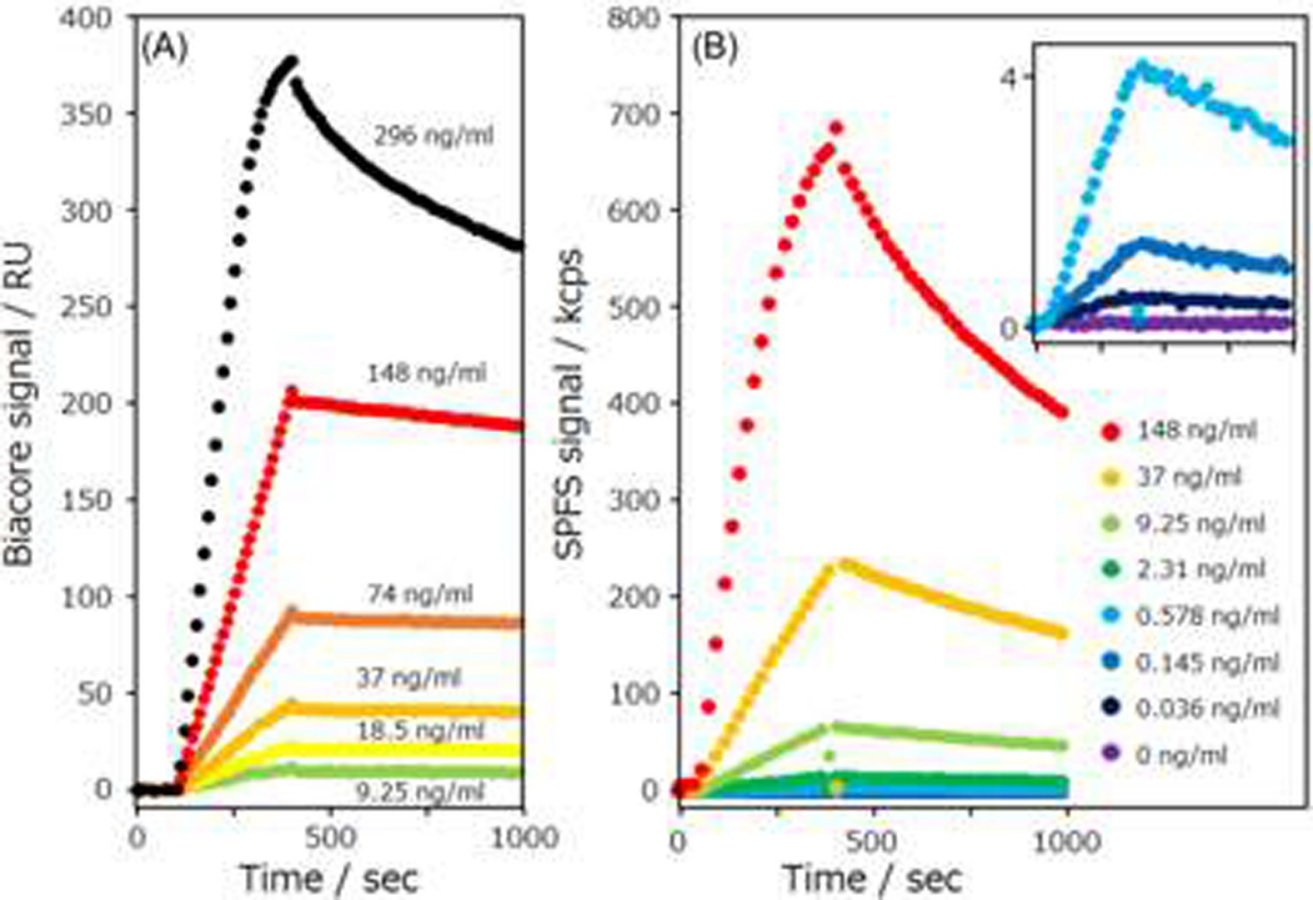
Affinity curves of wild-type anti-lysozyme sdAb. (A) Time course curve of Biacore system and (B) GC-SPFS fluorescence signal for the difference between the p- and s- polarizations with the lysozyme immunoassay by using the Alexa-647-labeled wild-type sdAb.

Fig 5 shows the time course curves for the interaction between the lysozyme and the Alexa-647-labeled wild-type anti-lysozyme sdAb with the (A) Biacore and (B) GC-SPFS systems. GC-SPFS fluorescence signals were automatically determined from the difference between p- and s-polarized fluorescence signals to eliminate the effect of the bulk fluorescence noise signal before washing buffer injection. Additionally, GC-SPFS fluorescence signals were switched to only measure p- polarized fluorescence during wash buffer injection. All the time course curves represent typical responses, which increased on injection of the Alexa-647-labeled wild-type anti-lysozyme sdAb and decreased on injection of the washing buffer. The affinity response of the GC-SPFS system was observed at an Alexa-647-labeled wild-type anti-lysozyme sdAb concentration of 0.036 ng/ml, that was 100 times smaller than that of the Biacore system (9.25 ng/ml), which is a significant advantage for interaction analyses targeting valuable or small samples.

**Fig 6:**
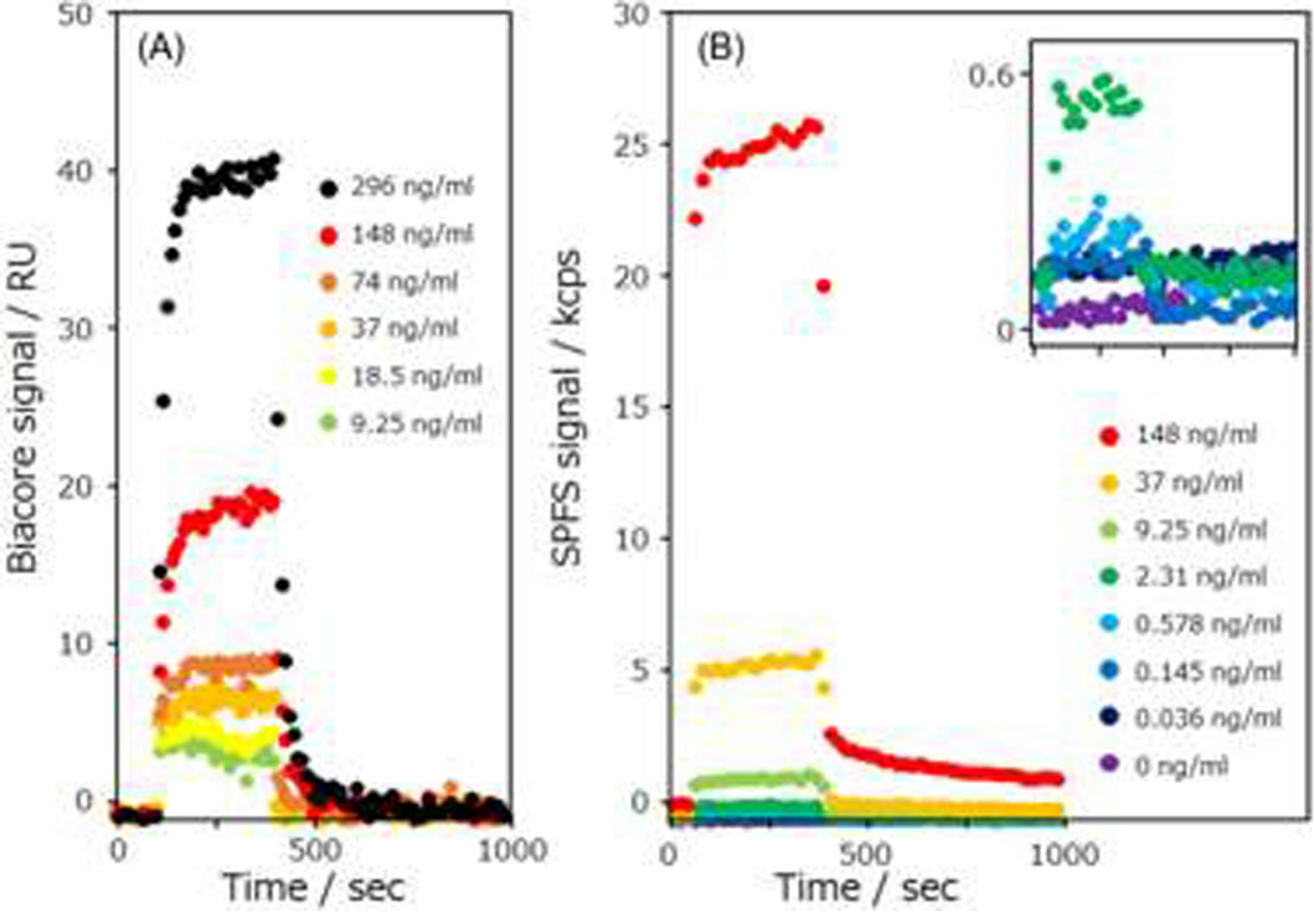
Affinity curves of anti-lysozyme sdAb Y52A mutant. (A) Time course curve of the Biacore system and (B) GC-SPFS fluorescence signal of the difference between the p- and s- polarizations with the lysozyme immunoassay by using the Alexa-647-labeled Y52A mutant.

Additionally, to determine the specificity of the GC-SPFS affinity analysis, we prepared the Alexa-647-labeled anti-lysozyme Y52A mutant as a low-affinity interaction model and conducted measurements with both the Biacore and GC-SPFS systems (Fig 6). We also calculated the kinetic affinities (k_on_, k_off_, and K_D_) of the wild-type anti-lysozyme sdAb and Y52A mutant using the BIA evaluation software (version 3.0 GE Healthcare Bio-Sciences AB) (see Table 1). We repeated the experiment of GC-SPFS system three times, and used fresh sensors and freshly prepared samples for each of them. The slight difference in k_on_ and k_off_ of Y52A mutant was observed between Biacore and GC-SPFS, we consider that this difference in sensor grams and analyses results may be due to differences in sensor surface, flow paths structure, and the concentration of sdAb. However, as there were no significant differences between the affinity kinetic K_D_ values obtained using the Biacore and GC-SPFS systems for both the wild-type anti-lysozyme sdAb and Y52A mutant, we verified that GC-SPFS is a new high-sensitivity interaction analysis technique exhibiting excellent correlation with the conventional method.

**Table 1:** Kinetic affinities of the wild-type anti-lysozyme sdAb and Y52A mutant.

sdAb type	Affinity value	Biacore2000^a^	GC-SPFS^b^
**Wild-type**	**k**_**on**_	2.79 × 10^5^ (0.08 × 10^5^)	6.70 × 10^5^ (0.09 × 10^5^)
	**k**_**off**_	4.23 × 10^−4^ (0.36 × 10^−4^)	9.24 ×10^−4^ (0.19 × 10^−4^)
	**K**_**D**_	1.52 × 10^−9^	1.38 × 10^−9^
	**Fitting conc.**	312.5pM ~ 40nM	2.4pM ~ 40nM
**Y52A**	**k**_**on**_	2.86 × 10^5^ (0.16 × 10^5^)	3.68 × 10^5^ (1.14 × 10^5^)
	**k**_**off**_	4.81 × 10^−2^ (0.005 × 10^−2^)	1.14 × 10^−1^ (0.04 × 10^−1^)
	**K**_**D**_	1.68 × 10^−7^	3.10 × 10^−7^
	**Fitting conc.**	2.5nM ~ 40nM	156pM ~ 40nM

Kinetic affinities (k_on_, k_off_, and K_D_) of the wild-type anti-lysozyme sdAb and Y52A mutant obtained using the Biacore 2000 and GC-SPFS systems with optical B/F separation.

^a^ Biaocre2000 analysis result of a measurement, the brackets indicate standard error. Standard errors of the Biacore software fitting indicated in parentheses.

^b^ GC-SPFS analysis result represents the median from three times measurements.

SPR and QCM technique are widely applied as label-free methods for investigating interfaces due to their sensitivity and ability to obtain real-time measurements [10]. Although GC-SPFS requires fluorescent labeling free of adverse effects from the target molecular conformation and interactions for accurate affinity measurement, it can provide highly sensitive real-time in-situ capability that cannot be achieved by conventional methods. Moreover, fluorescence analysis based on SPFS could be simply applied to multi-component and crude samples [22,23]. Recently, bioaffinity analysis techniques gained importance in next-generation drug screening, antibody medicine, and nucleic-acid medicine. Furthermore, interaction analysis of new biomaterials and medical devices under various conditions is important in guaranteeing the efficiency of biomaterial drugs [10].

### Comparison of the lysozyme sensitivities of the wild-type anti-lysozyme sdAb and Y52A mutant with a GC-SPFS immunoassay and ELISA

**Fig 7:**
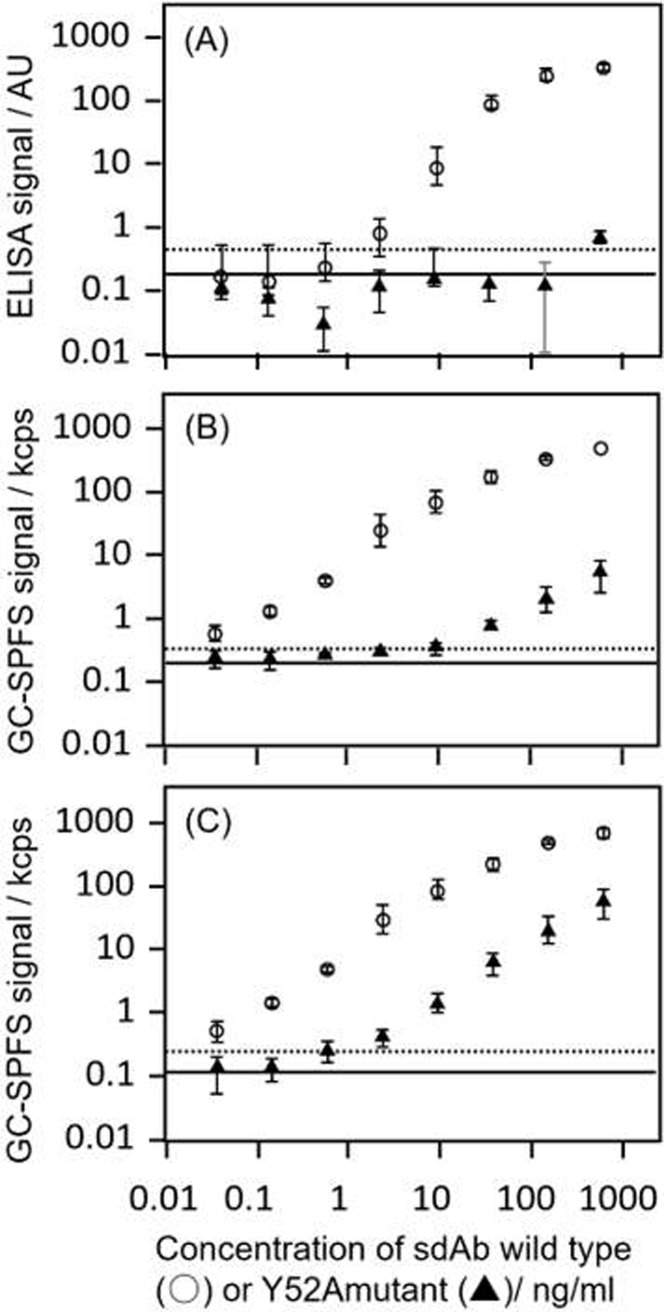
Calibration curves of the lysozyme immunoassay by using the wild-type sdAb (○) and Y52A (▲). (A) ELISA and GC-SPFS signals detected with (B) conventional washing B/F separation by measuring p-polarized fluorescence signals and with (C) optical B/F separation by measuring the difference between p- and s-polarized fluorescence signals. All the points were measured at three times. The dotted line represents the GC-SPFS assay blank with conventional washing B/F separation (mean = 200 cps, SD = 46 cps) and with optical B/F separation (mean = 113 cps, SD = 43 cps). The ELISA assay blank was 1.442 AU (SD = 0.461 AU).

Two recombinant proteins of sdAb that differ by only one amino acid residue to change the affinity property were utilized for precise immunoassay analysis. These recombinant proteins were effective to minimize the influence of molecular weight, surface charge state, and fluorescent labeling on analytical performance. Lysozyme-sdAb immunoassay curves obtained using the Alexa-647-labeled anti-lysozyme sdAb (○) and Y52A mutant (▲) with (A) ELISA, (B) GC-SPFS immunoassays with conventional washing B/F separation, and (C) with optical B/F separation are shown in Fig 7. The wild-type sdAb (○) calibration curves indicated that the detection limits (LODs: means of six replicates of the zero standard plus three standard deviations [SDs]) of (A) ELISA, GC-SPFS immunoassay with (B) conventional washing B/F separation and (C) optical B/F separation were 1.72, 0.03, and 0.02 ng/ml, respectively. With the high-affinity material assay condition like wild-type anti-lysozyme sdAb (**○**) (K_D_ value was analyzed 10^−9^ order in Table 1), there were clear differences in the sensitivity between the ELISA and GC-SPFS systems. However, there were no obvious differences between conventional washing B/F separation and optical B/F separation in the sensitivity and dynamic range in the GC-SPFS immunoassays. Nevertheless, the LODs of the Y52A mutant (▲) from the calibration curves of (A) ELISA, (B) GC-SPFS immunoassay with conventional washing B/F separation, and (C) with the optical B/F separation were estimated at over 1.0 μg/mL, 9.4 ng/ml, and 1.2 ng/ml, respectively. The deterioration in the LODs due to the decrease in the sdAb material affinity was confirmed as 100 times in ELISA and GC-SPFS with conventional washing B/F separation. However, the sensitivity degradation was strongly suppressed by using the optical B/F separation technique with the low-affinity material assay (K_D_ was of the order of 10^−7^ ~ 10^−6^ as shown in Table 1). These results indicated that GC-SPFS immunoassay with optical B/F separation could realize a cutting-edge of high sensitivity immunoassay that does not require significant material affinity to achieve good system sensitivity.

Generally, while almost all conventional high sensitivity methods need to relax the washing conditions to perform a low-affinity material assay, both higher sensitivity and a protocol with less washing are required for the true solution to the ADA problem. Optical B/F separation with the GC-SPFS method can also measure the specificity of low-affinity biomaterials, e.g., lectin, which are difficult to evaluate using conventional B/F washing.

In detecting carbohydrate chains on protein molecules using conventional antibodies, the carbohydrate chains of the antibodies sometimes need to be cleaved. However, cleavage decreases the antibody activity. Since sdAb antibodies prepared by phage display have no carbohydrate chains, they may be more suitable than traditional antibodies for use as capture ligands for glycoprotein immunoassays using lectin detection. We believe that the GC-SPFS technique using optical B/F separation will solve the problem of immunoassay and drug development in the near future.

## Conclusions

In this study, we applied an SPCE specification of the angular distribution and polarization by employing a metal-coated grating surface to distinguish sensor surface reactions from bulk fluorophores in a new optical B/F separation technique. When conducting more accurate analysis in biomolecular interaction, it is necessary to minimize its reactions as much as possible, but almost conventional analysis method cannot be accurately executed due to insufficient sensitivity under the low concentration and low affinity. Development of carbohydrate chain biomarkers is expected to increase significantly in the near future due to their disease specificity and genome important phenotype; however, their interaction is weak (K_D_ is of the order of 10^−7^ ~ 10^−5^ M), making quantification and affinity analysis are extremely difficult. GC-SPFS with the optical B/F separation technique was shown to be highly sensitive without the conventional washing B/F separation step, even for low-affinity materials. Even though SPR including BIAcore system is widly used in the field of ligand binding assay analysis as non-labeled measurement, we predict that GC-SPFS with the optical B/F separation technique will provide a number of novel opportunities to re-evaluate low-affinity biomaterials not only anitbody medicine but nucleic acid medicine. Additionally, GC-SPFS will provide innovative screening methods for biomarker in drug development and companion diagnostic developlment as a basic principle, since the GC-SPFS method requires no optical prism for plasmon enhancement on a thin gold film.

## Supporting information

S1 Fig Flowchart/block diagram of the methodology of this study. Polarization of GC-SPFS signal were basically evaluated by using Allophycocyanin. And next, we designed two evaluation step for GC-SPFS with and without optical B/F separation, first affinity analysis performance and second quantitative ligand binding assay performance.
(TIF)
