# Supplementary figures and images for: Highly sensitive biomolecular interaction detection method using optical bound/free separation with grating-coupled surface plasmon field-enhanced fluorescence spectroscopy (GC-SPFS)
Source: PLoS One. 2019 Aug 1;14(8):e0220578. doi: 10.1371/journal.pone.0220578 (PMC6675060; doi:10.1371/journal.pone.0220578)

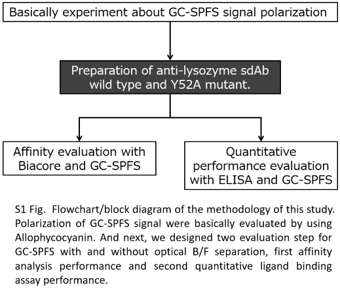

Supplement: S1 Fig — Polarization of GC-SPFS signal were basically evaluated by using Allophycocyanin. And next, we designed two evaluation step for GC-SPFS with and without optical B/F separation, first affinity analysis performance and second quantitative ligand binding assay performance. (TIF) [file pone.0220578.s001.tif]
